# Sustainable coffee: A review of the diverse initiatives and governance dimensions of global coffee supply chains

**DOI:** 10.1007/s13280-024-02003-w

**Published:** 2024-04-29

**Authors:** Dale R. Wright, Sarah A. Bekessy, Pia E. Lentini, Georgia E. Garrard, Ascelin Gordon, Amanda D. Rodewald, Ruth E. Bennett, Matthew J. Selinske

**Affiliations:** 1 ICON Science, School of Global, Urban and Social Studies, RMIT University, VIC 3000, Melbourne, Australia; 2 Department of Energy, Environment, and Climate Action, Arthur Rylah Institute for Environmental Research, Heidelberg, VIC 3084 Australia; 3 School of Ecosystem and Forest Sciences, University of Melbourne, Parkville, VIC 3010 Australia; 4 Department of Natural Resources and the Environment, Cornell University, Ithaca, NY USA; 5 Cornell Laboratory of Ornithology, Ithaca, NY USA; 6 Migratory Bird Center, Smithsonian’s National Zoo and Conservation Biology Institute, Washington, DC 20013 USA

**Keywords:** Coffee, Farmers, Governance, Literature review, Power dynamics, Sustainability

## Abstract

With a global footprint of 10 million hectares across 12.5 million farms, coffee is among the world’s most traded commodities. The coffee industry has launched a variety of initiatives designed to reduce coffee’s contribution to climate change and biodiversity loss and enhance the socio-economic conditions of coffee producers. We systematically reviewed the literature on the sustainability and governance of coffee production and developed a typology of eleven sustainability initiatives. Our review shows that coffee sustainability research has focused primarily on the economic outcomes of certification schemes. The typology expands our knowledge of novel sustainability initiatives being led by coffee farming communities themselves, allowing for an improved consideration of power dynamics in sustainability governance. Sustainability initiatives governed by local actors can improve sustainability outcomes by empowering local decision makers to assess direct risks and benefits of sustainable practices to the local environment, economy, and culture.

**Supplementary Information:**

The online version contains supplementary material available at 10.1007/s13280-024-02003-w.

## Introduction

Human activities continue to drive unprecedented changes in ecosystems and their ability to deliver the goods and services upon which humans depend, with recent research indicating that we have transgressed six of the nine planetary boundaries (Rockström et al. 2009; Richardson et al. 2023). The food system has potentially surpassed the safe planetary boundaries for two major axes of impact—biodiversity loss and phosphorus pollution—and poses significant planetary risks through its greenhouse gas emissions and overuse of fresh water and cropland (Rockström et al. 2020). It is therefore critical to address the impacts of agriculture and develop approaches that can shift food production systems towards a more sustainable trajectory.

Coffee is one of the top ten global commodities, farmed across 10 million hectares on about 12.5 million coffee farms (ICO 2014). However, the coffee sector has been challenged by a series of issues that threaten its social and environmental sustainability (Bacon et al. 2008). Historically, a series of global contracts, known as the *International Coffee Agreements*, were adopted to ensure the price stability of coffee and manage supply (ICO 2014). However, in 1989, the dismantling of the agreements alongside rapid growth in coffee supply from Brazil and other coffee-producing countries precipitated a price crash of almost 75%, which continued towards an all-time low of $0.50 per pound in 2001 (ICO 2014; Rueda et al. 2017). Increasing incidence of coffee leaf rust and other diseases have also plagued the sector in recent times (ICO 2014), while climate change is reducing the area suitable for cultivation (Lara-Estrada et al. 2021). Increasing recognition of the challenges facing the coffee sector has resulted in the development of a number of sustainability initiatives intended to address impacts of climate change, improve natural resource management and enhance the socio-economic conditions of coffee producers (Panhuysen and Pierrot 2020).

A theory of change is an explicit representation of the steps required to achieve a particular goal, usually starting from a long-term outcome, and subsequently identifying the actions and enabling conditions required to achieve that goal (Taplin and Clark 2012). The theory of change underpinning most sustainability initiatives or supply chain interventions is rooted in adjusting the behaviour of relevant actors or groups of actors within a supply chain (Newton et al. 2013). In recent decades, several supply chain policies, also known as voluntary sustainability standards or certifications, have been developed to trace and reduce the impacts of crops on biodiversity (Garrett et al. 2021). Coffee has been a focal commodity both for the development of certifications and for research regarding the outcomes of such certifications (DeFries et al. 2017; Tayleur et al. 2018; Traldi 2021). Alongside standards or certifications other sustainability initiatives being implemented include private corporate social responsibility programmes and government technical support programmes. These sustainability initiatives operate at various spatial and temporal scales and aim to address different challenges in the coffee production system, for example, targeting fair compensation for farmers, or ensuring environmentally sustainable practices. Sustainability approaches vary across a number of axes and may be defined either by the types of actor that are engaged, or how strict the requirements are for receiving the certification (Rueda et al. 2017). Alternatively approaches might be characterized by the level of involvement of the private actor, with some choosing “hands-on” policies and actions in direct relationships with coffee producers and the supply chain, whilst others act through pre-existing certifications in a more “hands-off” manner (Bager and Lambin 2020).

Alongside social and economic outcomes, voluntary sustainability standards and other sustainable or regenerative agriculture approaches are assumed to provide a number of environmental benefits including: improving soil health, reducing conversion of natural ecosystems, reducing pollution and off-farm ecological impacts, and improving the conservation value of the production landscape itself (Milder et al. 2014, Newton et al. 2020). Essentially, standards attempt to address a triple bottom line for sustainability, by encouraging positive environmental, social and economic outcomes (Garrett et al. 2021; Traldi 2021). Synthesis of the research regarding certifications has produced mixed findings; some case studies found positive outcomes for livelihoods and conservation (Garrett et al. 2021), whereas in other reviews the evidence of benefits was inconclusive (DeFries et al. 2017). A review focused on the economic effects of sustainability standards found that certifications boosted both the price paid for beans and household income (Meemken 2020).

Beyond certifications there is a wide range of initiatives being implemented collaboratively by multiple actors across the coffee supply chain (Millard 2017; Samper and Quiñones-Ruiz 2017; Bager and Lambin 2020). While diverse governance structures exist within coffee sustainability initiatives, most research has focused on certifications, with less consideration of other types of initiatives, their strengths or limitations, and their potential to create change (Samper and Quiñones-Ruiz 2017). Thus, the intention of this review is to document and characterize the diversity of sustainability initiatives and governance structures being applied throughout the coffee supply chain. Here, we define a sustainability initiative as: any stakeholder initiative introduced into, or developed within, a socio-ecological system, and intended to generate positive social, economic, and environmental outcomes within that system (Smith 2007; Newton et al. 2013). We focus on initiatives that apply to the production side of the supply chain, thus contributing to sustainability of the coffee growing landscape and its environment, and the socio-economic sustainability of coffee producers.

The diversity of sustainability initiatives in the coffee sector may create confusion for producers, retailers and consumers seeking to build and demonstrate market support for sustainable practices. To assist in navigating this complexity, we produce a typology of the various sustainability initiatives in operation to assist stakeholders in selecting an approach that fits their context and purpose. This typology informs a comparative analysis of the initiatives, focused on the defining characteristics of each, the actors, and scales at which they operate. The typology expands on previous work investigating coffee sustainability through the identification of diverse initiatives being led by coffee farmers themselves. This allows for an improved consideration of issues such as power dynamics in sustainability governance and reveals the bias in research and practice towards sustainability initiatives led by other supply chain actors, particularly those from upper-income and coffee-consuming countries. We also assess the outcomes being investigated, and the evidence base regarding the social and environmental outcomes produced by these initiatives. Research gaps are identified and contribute to a research agenda to support sustainability in the coffee supply chain.

## Methods

**Fig. 1:**
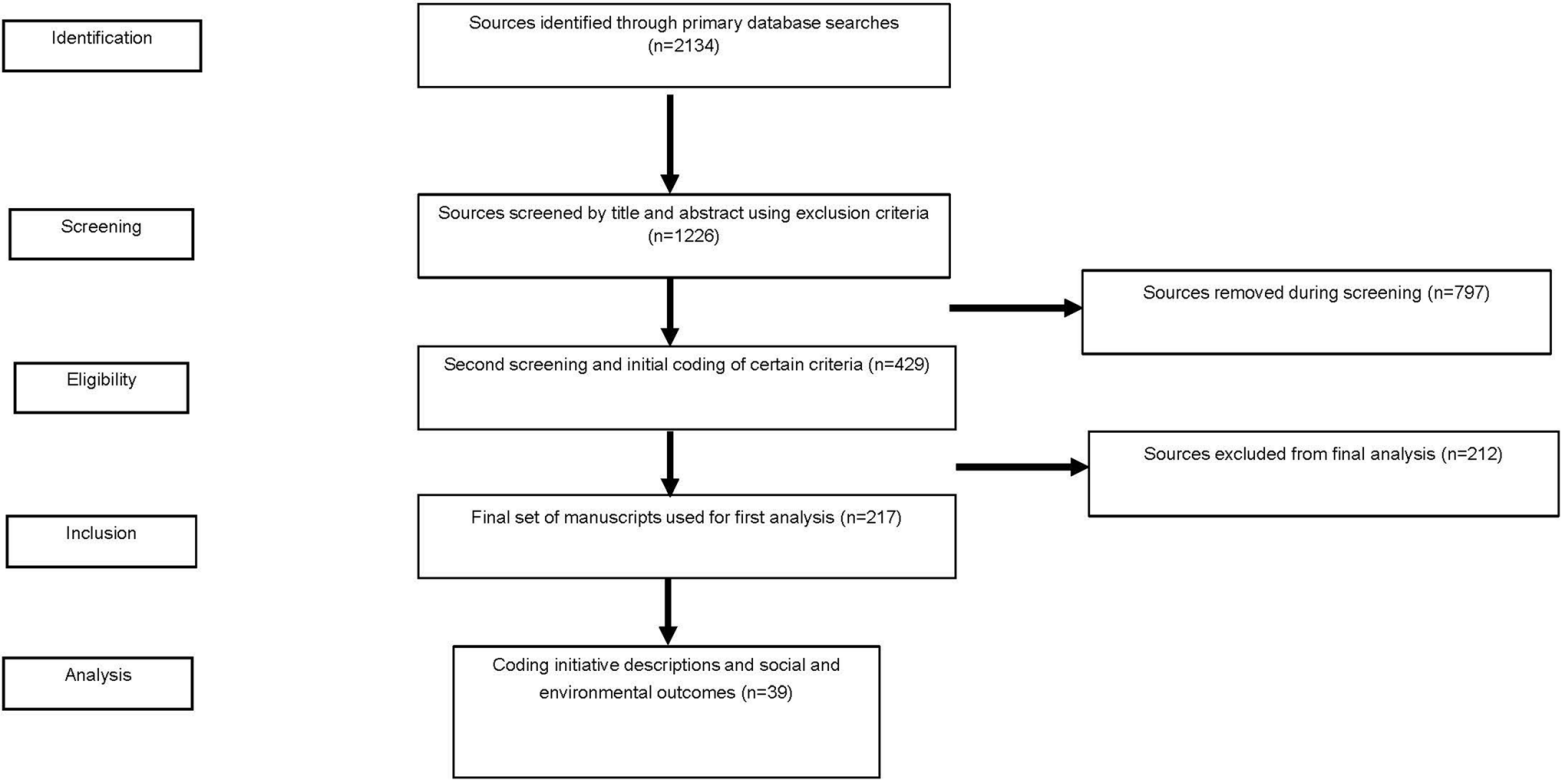
Systematic review protocol

In this review, we followed the RepOrting standards for Systematic Evidence Syntheses (ROSES) protocols, which were developed specifically for the conservation and environment sectors (Haddaway et al. 2018). These protocols identify the kinds of information that should be presented in a standard systematic review or systematic map. The major steps and information suggested in this protocol align closely with the stages of our review as described below and indicated in Fig. 1. We identified an initial set of 35 synonyms related to the research based on a pilot search of the relevant literature and our existing knowledge (SI 1). A naïve search string was constructed using a subset of these synonyms and used to generate an initial database of references from Web of Science. The *litsearchr* package in the R statistical analysis programme (2021) (Grames et al. 2019) was then used to scan the contents of this literature database for potential additional keywords, to further revise the search string (SI 1). The search string was iteratively tested, by adding and removing words and checking how this affected the total number of references identified, resulting in the final search string used for the review (Table 1).

**Table 1:** Final search string used for the review. The table highlights the different elements being targeted in the literature

Key topic		Environment and sustainability terms		Governance mechanisms		Aspects addressed
*Coffee*	AND	(biodivers* OR sustainab* OR environ* OR *forest*)	AND	(“*supply chain*” OR “*value chain*” OR certificat* OR *governance* OR polic* OR *instrument* OR *impact* OR *actor* OR *practice* OR *initiative*)	AND	(*social* OR *economic* OR *cultural* OR *power* OR *equity*)

We applied a topic search using this search string to a range of databases (Web of Science [topic search], Scopus [Title, abstract, keyword search], and ProQuest [Abstract search, limited to peer-reviewed literature]) for English language studies with no specified date range. The three databases were searched in February 2022, and the resulting 2134 citations were combined in R using the *revtools* package (SI 2) (Westgate 2019). These citations were imported into the online Covidence platform, which was used to identify and extract duplicates using a title, year, volume, and author matching algorithm (Covidence 2022). A subset of 60 identified duplicates were manually examined to ensure they were duplicates. This process resulted in a final dataset of 1226 publications; we reviewed the title and abstract of each, excluding publications that failed to explicitly mention coffee and those that focused on pure ecological, agricultural, or food sciences. We also excluded publications that focused solely on coffee quality, plant breeding and genetics, marketing, branding, or consumer psychology (see SI 3 for exclusion criteria). Consistency was checked by examining a random subset of 5% of the excluded papers. Application of the exclusion criteria resulted in the retention of 429 manuscripts for further review (Fig. 1), which were imported into Microsoft Excel (2018) for further screening and analysis.

At this stage, eligibility screening was carried out by reviewing the title and abstract of each manuscript again and checking additional manuscript text if further clarification was required. Because we were interested in the sustainability of coffee production, as opposed to consumption, only studies of initiatives in coffee-producing countries and regions were retained. Studies that considered other stages along the supply chain were also excluded, as were book chapters and other systematic reviews, to prevent double counting of outcomes. Although our study explicitly highlights initiatives focused on the production end of the supply chain, our analysis still considers the links between the different stages of the supply chain and how actors at various stages influence sustainability outcomes throughout the supply chain. During the eligibility screening the lead author documented any type of sustainability initiative encountered in any study, and the term used to describe it. He continued to add new terms and types of initiatives throughout this process of inductive coding, resulting in the preliminary table of sustainability initiatives (SI 6). This extensive list was characterized by similarity, and the different types were grouped to develop the initial list of 11 candidate sustainability initiatives. Additional exclusion principles were applied at this stage (SI 4), resulting in a final dataset of 217 manuscripts (SI 5) being retained for the first analysis (Fig. 1).

For each of the 217 manuscripts, information related to the type of sustainability initiative was documented, based on the candidate 11 types we had identified. We did not identify any additional, novel types of initiatives beyond those already identified in the eligibility screening, which resulted in these 11 candidate initiatives being retained as our final typology (Table 2). For each study, we also recorded whether the outcomes of a sustainability initiative were described, and the kinds of outcomes (social, environmental, economic) reported. The focal country of the study was documented, including multiple countries in some instances. The information extracted in this way comprised the first stage of analysis in our study (Fig. 1; Inclusion).

**Table 2:** Sustainability initiative typology

Initiative type	Definition	Key references	Agents	Scale of agents	Target group
Community & Cultural Initiatives	Community-led landscape management and community-based agritourism programmes which are aligned with the socio-cultural values of coffee cultural landscapes	(Candelo et al. 2018; Prihayati and Veriasa 2021)	Producers, Cooperatives	Local, Regional, National	Producers, Cooperatives
Producer Cooperatives	A voluntary association of people united by their common economic, social, or cultural goals in a co-owned and democratic venture	(International Cooperative Alliance 2023; Melo Torres et al. 2017)	Producers, Cooperatives	Local, Regional	Producers, Cooperatives
Agroecology programmes	Programmes of agricultural production involving the use of agroecological principles for farming, either initiated by coffee farmers themselves or with support from government or NGO programmes	(Häger et al. 2021; Le et al. 2021)	Producers, Cooperatives, (Government, NGOs)	Local, Regional	Producers, Cooperatives
Climate–smart agriculture programs*	Initiatives focused on enhancing the resilience of agricultural landscapes to the impacts of climate change, through financial and technical support for adaptation and mitigation strategies	(Bro et al. 2020)	Government, NGOs	National, Regional	Producers, Cooperatives
Geographic or Source Labels	Explicit legal or regulatory recognition of a source area for a specific product and product labelling to indicate that specific origin (and attributes such as quality or reputation associated with that geographic area)	(Hoang and Nguyen 2019; Marie-Vivien et al. 2014)	Government	National, Regional	Producers, Cooperatives, Consumers
Government Policy^	Government-led policies or programmes, often involving financial and technical support to farmers to deliver positive environmental and social benefits in a particular region	(Morais and César Pinheiro Da Silva 2021; Taringana and Mtisi 2019)	Government	National	Producers, Cooperatives
Payments for Ecosystem Services	Farmers or other landowners receive payments or other financial incentives for sustainable land management which ensures the delivery of ecological services such as water recharge and carbon capture for other beneficiaries	(Castro et al. 2013)	Government, NGO, Business	Global, National	Producers, Cooperatives
Relationship Coffee Model	Roasters, retailers, and smallholder coffee producers establish a long-term, direct trading partnership to ensure supplies of high-quality coffee in a mutually beneficial relationship	(Hernandez-Aguilera et al. 2018; Weber and Wiek 2021)	Business	Global, National	Producers, Cooperatives,
Corporate Social Responsibility^#^	Internal company commitments, standards and practices focused on coffee production processes, including environmental impacts, social concerns, quality, and traceability	(Bager and Lambin 2020)	Business	Global	Producers, Cooperatives
Certifications	Voluntary sustainability standards established by NGOs or other third-party actors which describe a set of specific economic, social, environmental and governance criteria for coffee production, often in exchange for a price premium	(Dietz et al. 2021)	NGOs	Global	Producers, Cooperatives, Consumers
Global multi-actor initiatives	Cross-sector partnerships and platforms comprising voluntary commitments aimed at improving economic, social, and environmental outcomes for coffee sector stakeholders	(Manning and von Hagen 2010; Sustainable Coffee Challenge 2020)	NGOs, Business, (Cooperatives)	Global	Producers, Cooperatives, Retailers

The typology provides a basic definition of each initiative, key references that describe it in further detail, the agents who primarily lead the design and implementation, the scale across which they function, the target group of actors responsible for changing behaviours, and how each initiative type aligns with the IPBES policy instrument categorization (IPBES 2019)

^*^Whilst agroecology programmes address environmental outcomes more broadly, Climate Smart Agriculture initiatives always have an explicit focus on adapting to climate change

^Whilst Payments for Ecosystem Services are often implemented as part of a government policy, we distinguished them from other policies related to financial and technical support from the relevant government departments

^#^CSR initiatives may also be considered as first-party certifications, where the business designs, implements, and audits their suppliers against their own company standard. Examples include the Nespresso AAA or Starbucks CAFÉ standards. These are distinct from the third-party approaches which we classify as Certifications, in which an external group designs a standard and assesses other entities against this standard, such as the Rainforest Alliance or Smithsonian Bird-Friendly certifications

The next step in the analysis consisted of reviewing full manuscripts for outcomes and extracting additional information to inform our typology. Because other recent reviews assessed primarily economic outcomes (DeFries et al. 2017; Meemken 2020), we focused only on those studies that examined both social and environmental outcomes (*n* = 39; Fig. 1), as identified during the previous step. Whilst we do not explicitly code and quantify the economic outcomes from these initiatives as part of our results, our analysis does nonetheless consider economic factors in relation to the overall sustainability of coffee production. For each manuscript, information pertaining to predefined themes was coded using Nvivo 12 Plus (2018). Our review drew on classifications of policy instruments from the literature, to identify ways that we could characterize the sustainability initiatives (Schneider and Sidney 2009; Rueda et al. 2017; Coffey et al. 2022). We extracted text which related to the actors, goals, scale, defining characteristics, and a theory of change for each initiative, themes which provided the information required for our typology.

Our typology drew an important distinction between *target groups* as the “peoples, groups and organizations” whose behaviour is intended to change and *agents* as the actors “who are assigned authority (or take the lead on) developing and implementing an initiative” (Coffey et al 2022). A similar approach was applied by Rueda et al (2017) who organized sustainability instruments in the agri-food sector according to their scope or “the supply actor bound by the instrument”. This corresponds to target groups in our framing. In addition to the information required for the typology, we also extracted any text describing power dynamics among actors, by examining text including aspects of equity, agency, and inclusive participation of actors within initiatives in the coffee supply chain. The statements relating to power dynamics were subsequently analysed and grouped based on their similarities. Actors were grouped as “downstream” or “upstream” relative to each other within supply chains, with producers and cooperatives occurring downstream of NGOs, government-level initiatives, and sourcing or trading companies. We extracted any text that described the relationship among actors at different levels and used it to classify each relationship as “creating a power imbalance”, “maintaining equity”, or “addressing a power imbalance” based on which actor drove the sustainability initiative. Actions where upstream actors conceptualized, required, and/or directed the implementation of a sustainability initiative were considered to be “creating a power imbalance”. Relationships where both actors achieved consensus or contributed equally to decision making were considered “maintaining equity”. Relationships where the downstream actors were empowered and supported to set sustainability priorities and develop implementation methodologies were considered “addressing a power imbalance”.

We drew on the Sustainability Assessment of Food and Agriculture (SAFA) framework to guide our outcomes analysis, focused on the sustainability dimensions of environmental integrity and social wellbeing (FAO 2014). Good governance and economic resilience are additional sustainability dimensions but were not considered for this research. Environmental Integrity is characterized by six themes, namely Atmosphere, Biodiversity, Water, Land, Materials and Energy and Animal Welfare, and a variety of sub-themes for each. We coded statements related to the sub-themes for all dimensions except Animal welfare and Materials and Energy. Social wellbeing includes six themes: Decent livelihoods, Fair-trading practices, Labour rights, Equity, Human safety and health, and Cultural diversity. Any outcomes related to the sub-themes were categorized accordingly. Outcomes were identified by reviewing the results or findings sections of the relevant papers. Because studies varied widely in how outcomes were measured, we could not compare the effectiveness of initiatives using quantitative measures. Instead, we used a vote counting approach, recording the individual outcomes in a study as positive, negative, or inconclusive based on the direction relative to any baseline in the study. In studies that included multiple individual outcomes, each outcome was included separately. Quantitative or qualitative outcomes from the implementation of an initiative and perceived or modelled benefits were coded in this way. For the purposes of this review, different outcomes in each study correspond to different cases.

A final step of the analysis involved grouping the sustainability initiatives in our typology based on the primary agents of each initiative and how far removed they were from the focal coffee growing landscape and coffee producers. By analysing our findings according to these scales of governance, we sought to determine whether the distance between the agents and the target group or landscape influenced the delivery of outcomes. There are limitations to applying a vote counting approach (Haddaway et al. 2020); however, our intention is not to indicate which are the most effective types of initiatives, but rather to illustrate the state of the evidence base and identify potential gaps in the monitoring of outcomes.

## Results

**Fig. 2:**
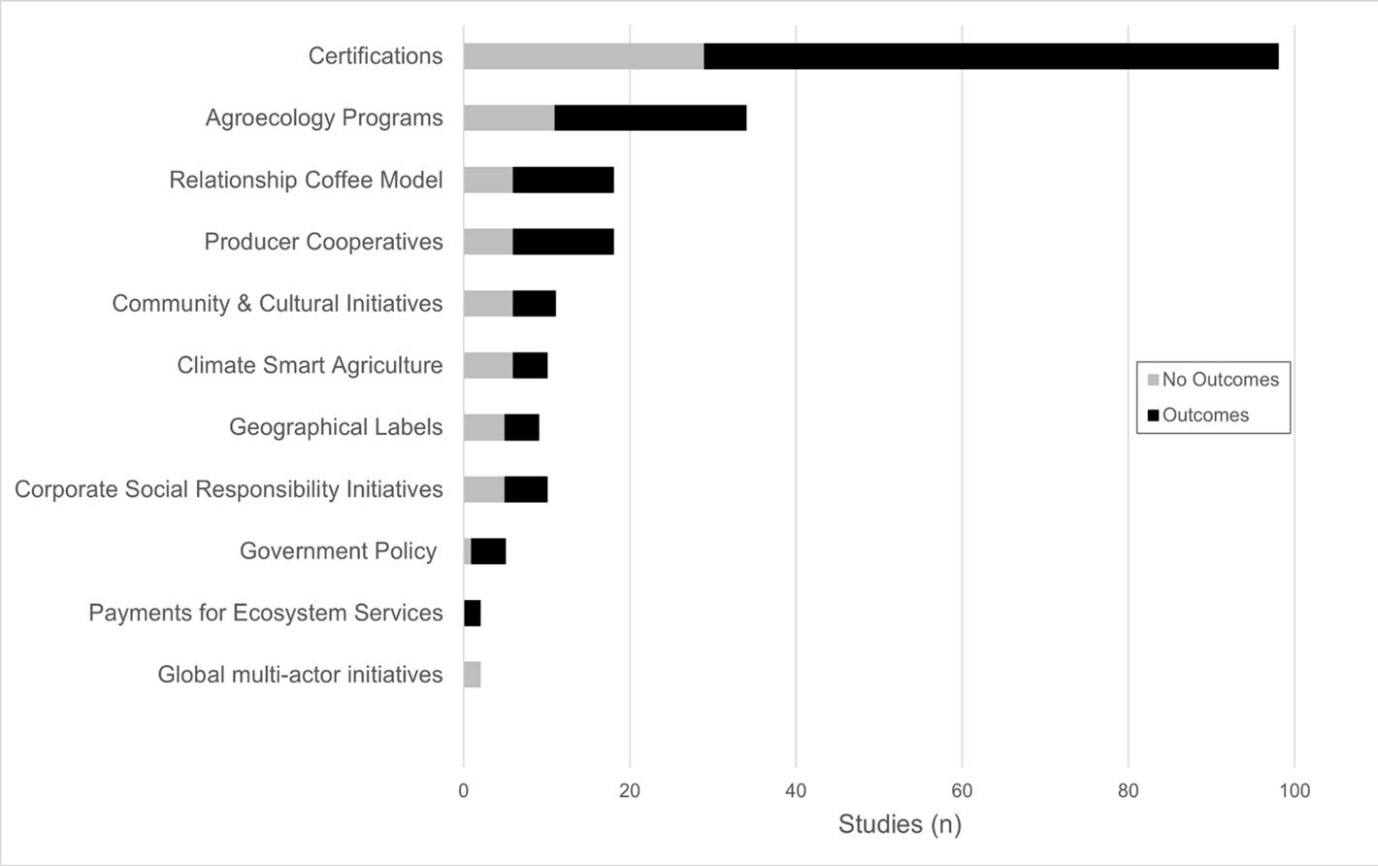
The number of studies for each type of sustainability initiative in the coffee supply chain, indicating they did (black) or did not (grey) document social, environmental, or economic outcomes

Following an extensive screening of the literature, our review documented a diversity of sustainability initiatives in the coffee sector, with almost 40 different terms being used (SI 6). Our typology discerned 11 types of sustainability initiatives in use across various stakeholders in the coffee sector (Table 2). This comparative typology then informed our subsequent analysis of the governance and outcomes of the sustainability initiatives. Based on our classification of the studies that documented outcomes of initiatives during our first analysis (Fig. 1—Inclusion, *n* = 217), it was clear that certifications or voluntary sustainability standards were the most well-studied group, comprising nearly half of our dataset (*n* = 98, Fig. 2). Those studies that did not measure outcomes were mostly concerned with politics and governance aspects of sustainability initiatives.

**Fig. 3:**
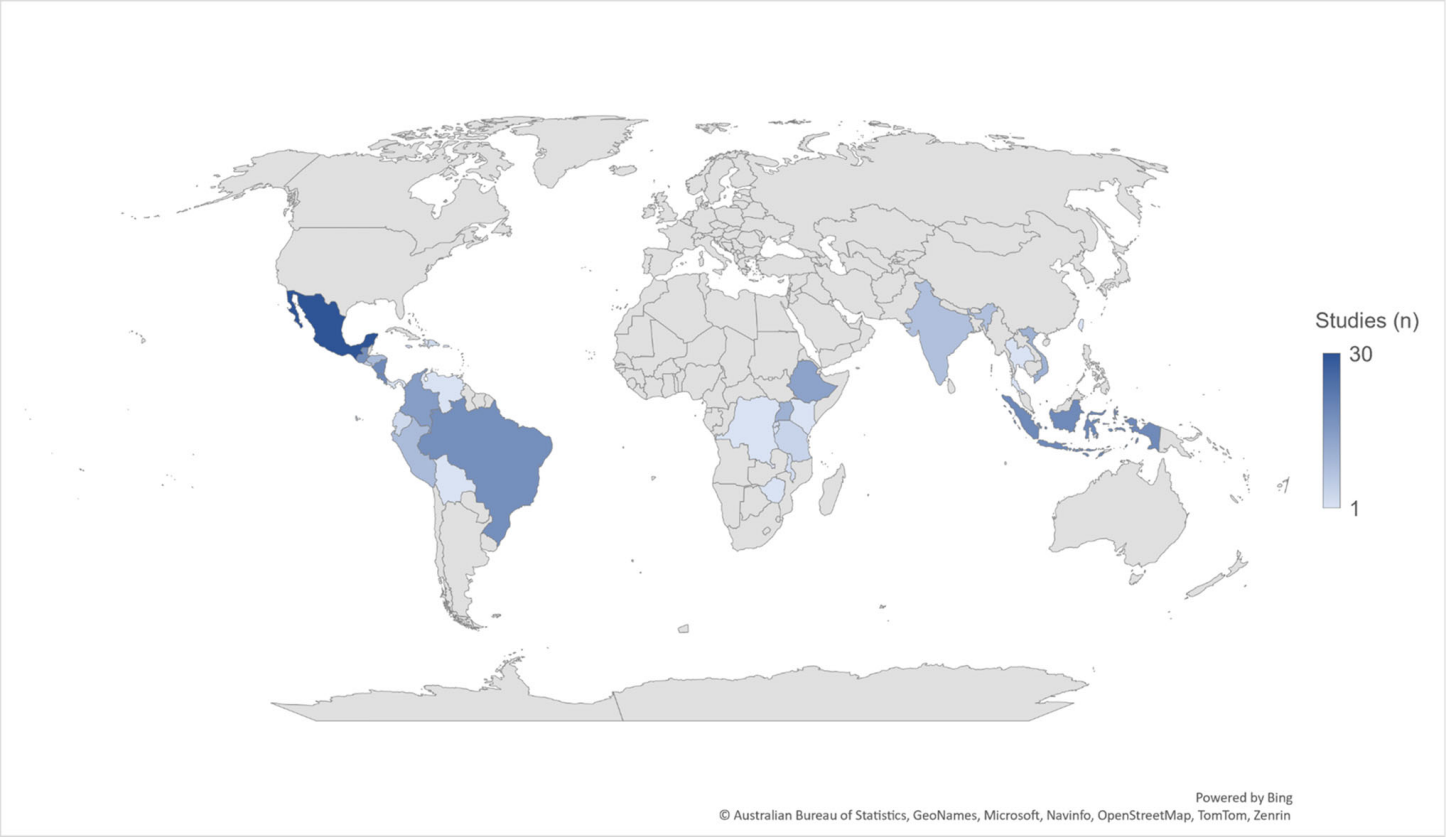
Global distribution of studies investigating sustainability initiatives in the coffee supply chain included in our dataset (*n* = 217)

**Fig. 4:**
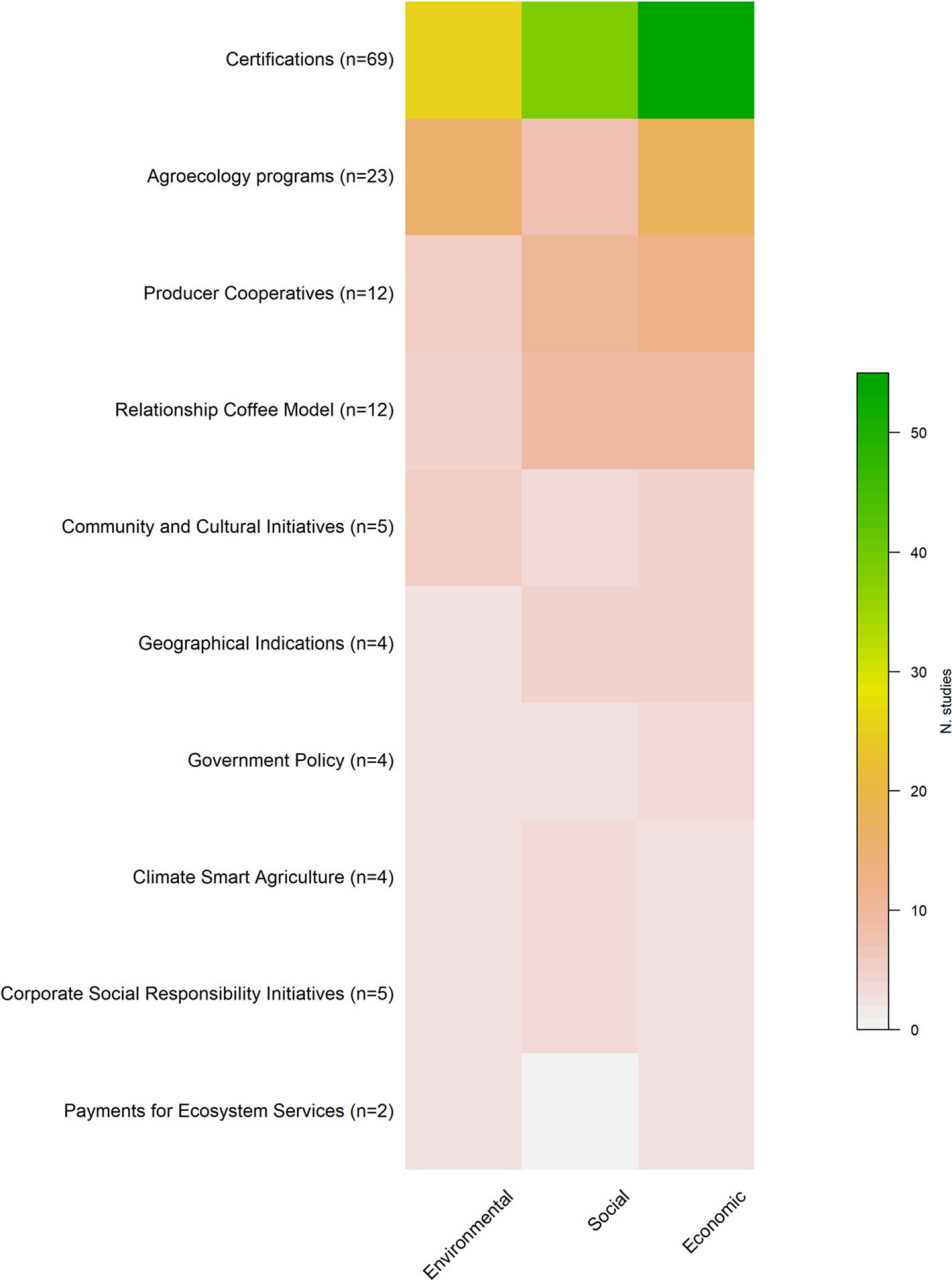
The number of studies investigating environmental, social and / or economic outcomes of coffee sustainability initiatives (*n* = 140), ranked from those with the highest number of outcomes to the least. A single study might examine all three types of outcomes

Sustainability research in the coffee supply chain covers the major coffee production countries and regions, with a bias towards research in the Americas, and particularly Mexico (*n* = 30, 14%) (Fig. 3). This bias is disproportionately high when considered relative to coffee production in Mexico (approximately 2% of global supply, SI 7). Notably, there were no studies within our dataset on coffee sustainability initiatives in West African coffee-growing countries, including Côte d’Ivoire, Cameroon, and Gabon. Some studies investigated dynamics at a global scale (*n* = 31) and are not represented in Fig. 3. Of the 217 studies we reviewed, 65% (140) documented at least one social, environmental, or economic outcome. Economic outcomes were most frequently evaluated (*n* = 110, 79%), particularly for certification programmes (Fig. 4), followed by social (*n* = 80, 57%) and environmental (*n* = 65, 46%) outcomes. Many of the sustainability initiatives had fewer than ten studies examining any outcomes, with Payments for Ecosystem Services comprising the fewest studies (Fig. 4).

**Fig. 5:**
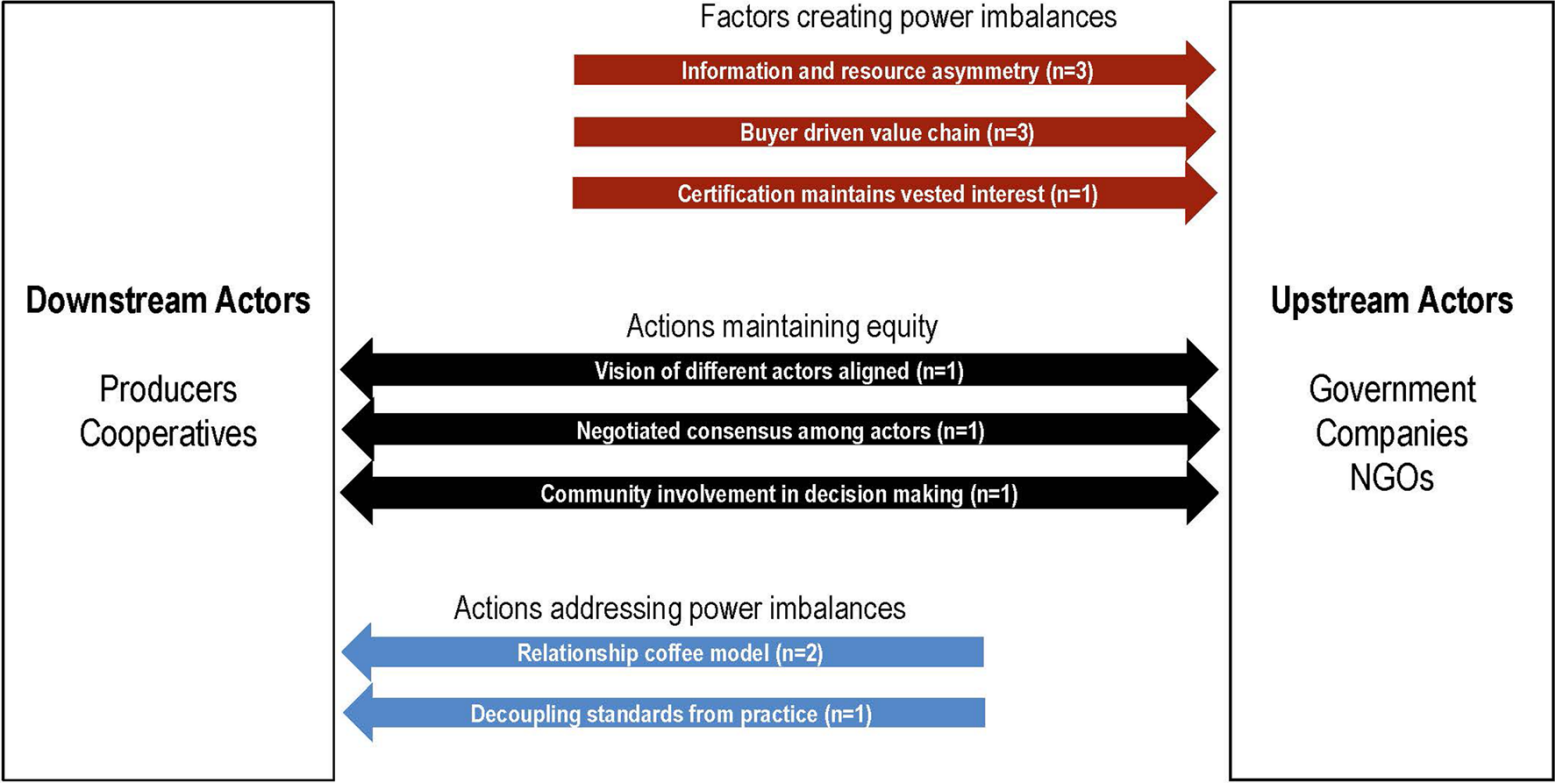
Power flows between different actors operating within the coffee supply chain. The sample sizes indicate the number of studies which included each factor or action

The statements we extracted relating to power dynamics mostly described relationships where actors higher in the supply chain exert power (*n* = 7), due to information and resource asymmetry and the nature of a buyer-driven value chain (Fig. 5). These dynamics act to maintain existing power asymmetry in the coffee supply chain. Fewer studies examined processes that improved equality between actors (*n* = 3), such as negotiating shared visions, building consensus, and/or ensuring democratic involvement of all actors (Fig. 5). A third power dynamic highlighted in three studies related to coffee producers’ attempts to tackle power imbalances, including through more equitable producer–retailer relationships emerging from the relationship coffee model (*n* = 2), and locally adaptive producer responses to certification requirements (Fig. 5).

### Scales of sustainability governance

**Fig. 6:**
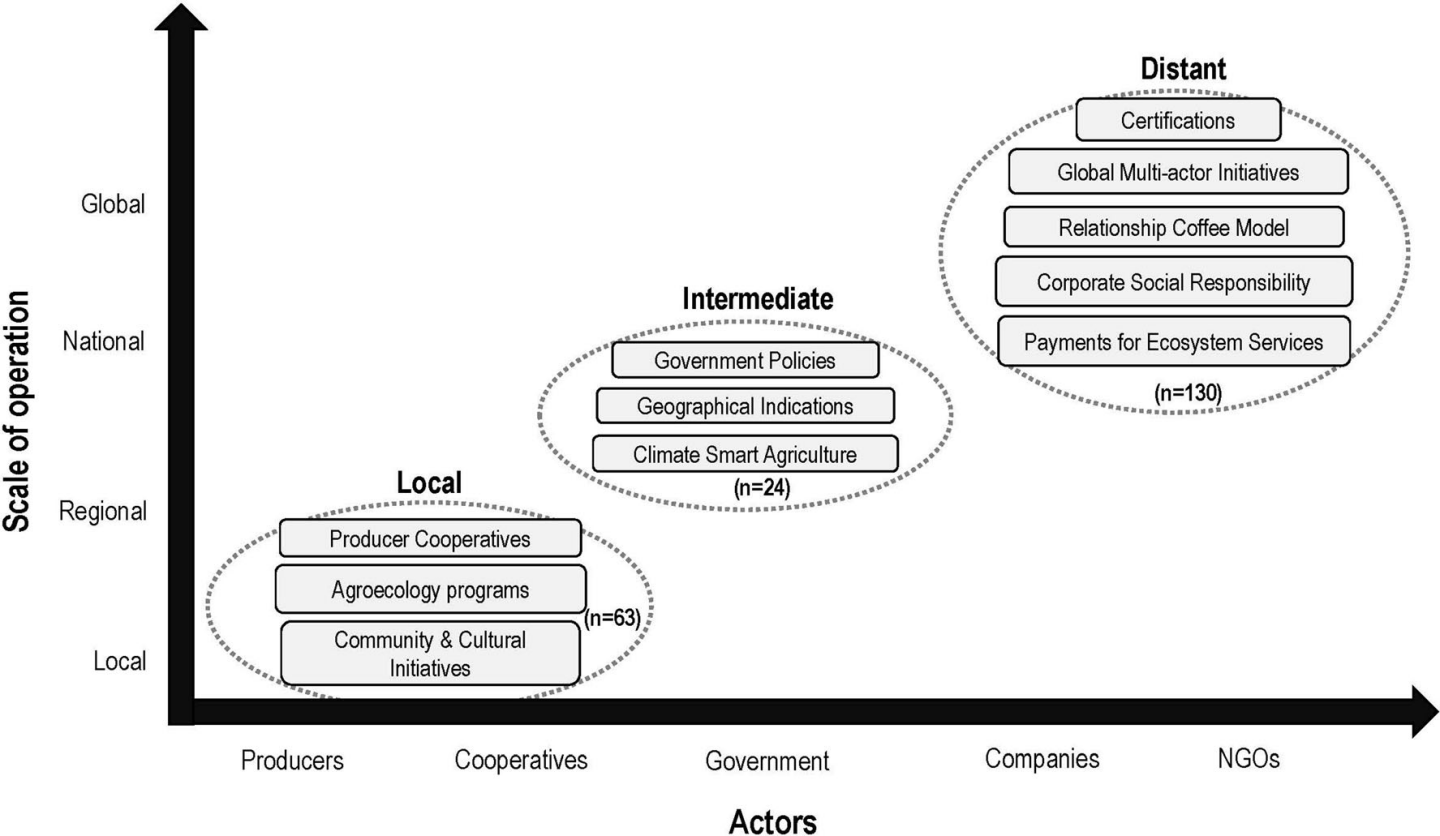
Scales of governance for sustainability initiatives in the coffee supply chain, showing the actors leading sustainability initiatives (agents, x-axis) and their scales of operation (y-axis). The total number of studies is indicated in brackets for each grouping

When we classified the central agents of each sustainability initiative (i.e. those who identify the actions to be undertaken by the target group) (Table 2), three broad groupings emerged (Fig. 6). Local initiatives, such as cooperatives and agroecology programmes, were designed and implemented by coffee producers themselves. Intermediate initiatives, such as geographic labels, were generally led by governments. Distant initiatives, such as corporate social responsibility programmes, certifications, or multi-stakeholder platforms, are designed and led by global actors. Whilst intermediate and distant initiatives are designed and led by actors located far from the coffee producing region, they all require action from stakeholders throughout the supply chain (for example, see *Target Groups* classification in Table 2).

The Relationship Coffee Model (RCM) occupies a unique space in this framing, by attempting to bridge both the physical distance and multiple stages of the supply chain, by focusing on direct-trade relationships between coffee producers and retailers (Hernandez-Aguilera et al. 2018; Edelmann et al. 2022). In these relationships, retailers and producers form partnerships which lead to the co-design of sustainability initiatives intended to meet the motivations of both parties. These are somewhat different from the other *Distant* initiatives, primarily designed by actors more removed from the production landscape. Whilst the RCM aims to bridge this distance, it is still enacted primarily at the discretion of coffee roasters or retailers, hence our placement of it as a *Distant* initiative, based on the lead agent location (Fig. 6).

### Sustainability initiative outcomes

Of the 39 studies that documented both social and environmental outcomes, we found 63 cases describing environmental outcomes (Table 3). In general, there was a lack of evidence for outcomes across most environmental outcome categories. The greatest number of positive outcomes related to the biodiversity and land categories, with a disproportionately high number of positive environmental outcomes (relative to the number of studies) documented for local level initiatives. We found no studies that evaluated the environmental outcomes of global multi-actor initiatives (Table 3). The documentation of negative environmental outcomes of certifications suggests that certain sustainability initiatives could have unintended consequences in some contexts or have insufficient power to alter negative environmental trends.

**Table 3:**
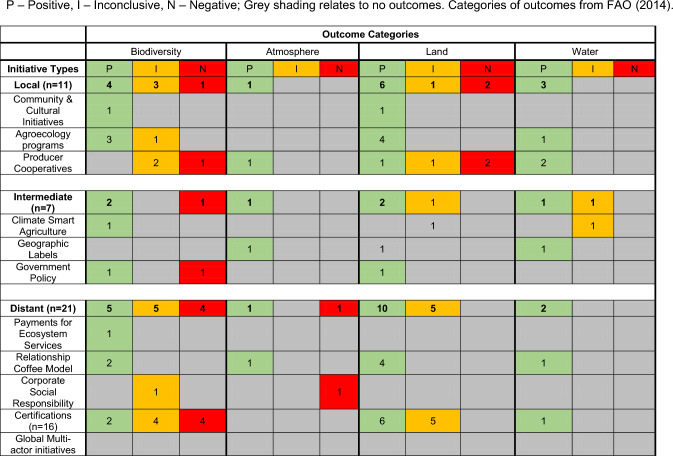
Classification of environmental outcomes documented for sustainability initiatives in our dataset (*n* = 39)

There was even less evidence for social outcomes delivered by the different initiatives, with only 46 cases across our social outcome categories (Table 4). Positive social outcomes were evident for local-scale initiatives and often included improving livelihoods through income diversification, establishing fair trade practices, protecting the health and safety of workers, safeguarding labour rights, and capacity building among local leaders. Agroecology programmes often focused on food security and income diversification to buffer against the volatility in coffee markets (Häger et al. 2021; Pronti and Coccia 2021). Community and Cultural Initiatives emphasized cooperation as a necessary element of a well-organized coffee agritourism initiative (Candelo et al. 2019). These initiatives also empowered coffee producers as independent entrepreneurs, who could then develop their own governance systems (Candelo et al. 2019; Prihayati and Veriasa 2021). Most positive social outcomes were focused on ensuring decent livelihoods for coffee producers rather than improving equity along the supply chain. We also found evidence of negative equity outcomes for those initiatives operating at intermediate and distant scales. For example, a Geographical Indication in Jamaica left coffee farmers with no influence over how the initiative was implemented, further reinforcing supply chain inequities (Francis et al., 2013). We found less evidence of negative outcomes than positive outcomes across both the environmental and social categories (Tables 3, 4). Three positive outcomes for Cultural Diversity were documented but are not included in Table 4.

**Table 4:**
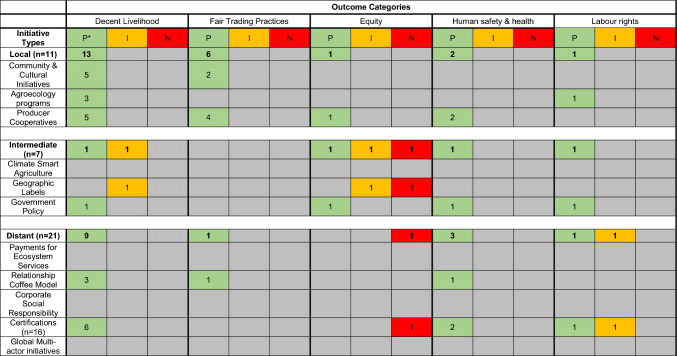
Social outcomes documented for sustainability initiatives (*n* = 39)

## Discussion

Through a literature review, we developed a typology of 11 types of sustainability initiatives operating in the coffee sector (Table 2). This typology resulted from a systematic screening of the relevant literature, which, to our knowledge, is the first comprehensive attempt at documenting the broad range of sustainability initiatives undertaken by all actors operating in the coffee supply chain. Other studies that have developed similar typologies have focused primarily on companies and corporate investments in coffee sustainability (Rueda et al. 2017; Bager and Lambin 2020). By highlighting the role of coffee producers in sustainability initiatives [for example, see the descriptions of Community and Cultural Initiatives, Producer Cooperatives and Agroecology programmes (Table 2)], this typology improves our understanding of key actors, such as farmers and their collective associations, which play a critical role in the sustainability of the coffee landscapes. Previous research, which has focused on actors in coffee-consuming, rather than coffee-producing, countries, may have inadvertently overlooked these important actors.

Our research illustrates the range of scales and actors involved, and the diversity of approaches being used, to pursue sustainability in the coffee supply chain. Whilst the diversity of initiatives is encouraging, research has focused predominantly on certifications and the Americas. Certifications were by far the most examined sustainability initiative, even though other approaches have also been around for 20 years or more. Unfortunately, we cannot determine the extent to which this pattern reflects a reporting or publication bias or a true predominance of certifications in sustainability initiatives in the coffee supply chain. Certifications naturally lend themselves to academic research as control and treatment groups can be readily evaluated, as can economic outcomes, which were the most assessed outcome category. This is important, because economic disparity is a core concern for coffee sector sustainability, especially for smallholder producers (Sachs et al. 2020). However, economic factors represent only one component of wellbeing (Stiglitz et al. 2009) which is characterized by additional social factors such as community cohesion, connection, trust, and personal development.

Compared to economic assessments, there have been fewer evaluations of social and environmental outcomes, limiting our ability to understand the effectiveness of sustainability initiatives. Whilst we did document evidence of positive environmental outcomes for most sustainability initiatives (Table 3), we were unable to conduct a comparative analysis of their effectiveness due to a lack of evidence and consistency in the literature. Future research should focus on comparing outcomes across the broad suite of sustainability initiatives, using standardized metrics where possible. Such metrics might include, for example, the default indicators suggested in the Sustainability Assessment of Food and Agriculture (SAFA) framework, which provides standardized metrics across multiple dimensions and themes related to agricultural sustainability (FAO 2014). Applying the indicators in that framework in the future research would facilitate comparisons of alternate initiatives and the outcomes they deliver.

### Novel and emerging sustainability initiatives

Our review identified the following novel and emerging types of initiatives to improve the sustainability of coffee: agroecology transitions led by farmers, community and cultural initiatives, geographic labels, and the relationship coffee model. Agroecology is a practice which applies ecological principles in the design of sustainable agricultural systems which also support the resilience and empowerment of smallholder farmers (Altieri 2002; Altieri and Toledo 2011). Agroecology programmes have historically been delivered as part of technical support projects for coffee producers from businesses, NGOs or government (Pronti and Coccia 2021). However, we documented novel agroecology programmes initiated by producers themselves to address environmental issues such as soil erosion or pollution from agrochemicals (Le et al. 2020; Häger et al. 2021), as well as to enhance livelihood security through diverse food products or income generated from the shade trees (Castro et al. 2013; Le et al. 2021; Urgessa Waktola and Fekadu 2021). Locally led agroecological initiatives have the potential to address power imbalances and deliver positive social and environmental outcomes, but to date have received less research attention. Future research could investigate the personal or community motivations behind such programmes or examine which structural and regulatory drivers either facilitate or hinder these transitions. Increasing global interest in regenerative agriculture as a key response to the climate and biodiversity crises could also be used to support agroecology transitions in practice (Newton et al. 2020). A key question in relation to these locally led initiatives is how actors at other stages in the supply chain can best respond to and support these farmer-led initiatives, without perpetuating the buyer-driven power dynamics of the coffee supply chain. Perspectives from intersectional environmentalism and political ecology could provide useful framing for such research, ensuring that complex ethical considerations are given proper attention (Robbins 2019).

Community and cultural initiatives differ from agroecology programmes through their explicit focus on community-based agritourism and community-based forest management as pathways to sustainability, and their consideration of the socio-cultural values that underpin coffee cultural landscapes. Community-based coffee tourism is a niche experience, which aims to expose tourists to working farms that use traditional, often agroecological, farming approaches (Candelo et al. 2019), and are usually embedded in broader coffee cultural landscapes (Yudhari et al. 2020). Coffee cultural landscapes are like Biosphere Reserve models, in which both natural and cultural values are promoted and conserved (UNESCO 2023). These approaches exist in Colombia (Martínez 2016), Ethiopia and Indonesia (Yudhari et al. 2020) as a method to conserve the biocultural diversity of these regions within a working production landscape. They were under-represented in our dataset and require further investigation, particularly as they exist at the intersection of social justice and environmental conservation, core concerns for transformative change (Visseren-Hamakers et al. 2021). Future research could examine the specific kinds of cultural and traditional ecological knowledge which are used to manage these landscapes, paying attention to issues of knowledge appropriation. Understanding how such knowledge is transmitted across individuals and generations, and how this might be affected by increasing rural emigration, is essential to supporting the longevity of these initiatives.

Geographic labels are commonly associated with products such as wine or champagne, where registration and labels protect the provenance of the product (Francis and Hyman 2013). They are usually linked to a physical location and its associated soils, climate, elevation or aspect (also described collectively as terroir), all of which in turn influence the product’s flavour profile (Bager and Lambin 2020). This is also true for coffee, and geographic labels are often associated with specialty, single origin coffee from specific farms or regions (Francis and Hyman 2013; Hoang and Nguyen 2019; Morais and César Pinheiro Da Silva 2021). Marketing and branding linked to these geographic labels can attract higher prices, potentially improving the economic outlook for smallholder producers in denominated geographies by capturing more value in the supply chain (Rueda et al. 2017; Samper and Quiñones-Ruiz 2017). Whilst these initiatives represent an opportunity for greater value capture, geographic labels enacted at national levels are prone to corruption and control by political elites, resulting in exclusion and inequitable trading relations for small-scale farmers (Francis and Hyman 2013). This leads to the question of whether geographical indications can be used to address power dynamics between exporters and importers of coffee, given their relationships are often already underpinned by the power dynamics between (for example) upper- and lower-income countries. It also calls into question the efficacy of these types of initiative in addressing within-country power imbalances between coffee farmers and other supply chain actors. Unfortunately, we do not know the overall prevalence or effectiveness of geographical indications in the coffee sector, apart from the information presented here.

The relationship coffee model, closely associated with a direct-trade model, seeks to shorten the supply chain by reducing the distance between coffee producers and consumers (Hernandez-Aguilera et al. 2018). This is intended to confer greater socio-economic benefits to the coffee producer by removing intermediaries and their associated costs. This approach is also being used by retailers and roasters who wish to better understand and address risks in their supply, as well as a broader recognition of addressing environmental impacts of commodity products. There are now many examples of similar producer-level support and engagement initiatives from coffee roasters around the world (Weber and Wiek 2021). Improved knowledge of how the relationship coffee model can drive sustainability transitions is required, particularly during these initial stages of its development. Such knowledge could include the motivations of both retailers and producers participating in these partnerships, and whether they are satisfied with the outcomes being delivered. Research may also ask whether these partnerships are perpetuating or challenging existing power dynamics in the coffee supply chain, and what the focal outcomes are, for example, whether retailers and roasters are more concerned with environmental conservation or the social and equity outcomes of sustainability.

### Power dynamics in the coffee supply chain

Coffee is traded in a buyer-driven supply chain in which large multinational companies, roasters and retailers have the potential to exert undue influence and capture significant value (Bager and Lambin 2020). The dismantling of the coffee trade agreements and liberalization of the coffee value chain exacerbated power asymmetries, benefiting multinational traders (Mithöfer et al. 2018; Grabs and Ponte 2019). Coffee producers received less than 10% of the total value in this supply chain in 2015 (Samper and Quiñones-Ruiz 2017; Bager and Lambin 2020). A critical component of these unequal power dynamics relates to the information asymmetry in the supply chain, where smallholder farmers have less access to the required knowledge and skills to engage in a global market (Candelo et al. 2019). For example, farmers interviewed in Jamaica described that they lacked any knowledge of how the coffee supply chain worked, beyond the stage of delivering their beans to the local processing facility (Francis and Hyman 2013). They also lacked trust in the government structures set-up to support them, had no representation in governance structures, and ultimately were unable to voice their concerns (Francis and Hyman 2013). Future research on coffee economics could seek to identify those instances in which there is more value captured by coffee farmers and understand the dynamics which support such outcomes. Buyer-driven value chains characterize many tropical commodities and research should examine where and how such inequities are being challenged to promote just transitions towards sustainability.

Certifications and sustainability standards are intended to act in support of coffee farmers, providing some leverage for them in acquiring higher prices or bargaining power for their product (Bager and Lambin 2020). Others have argued that certifications serve the vested interests of coffee buyers, due to their inability to meet farmer’s needs and limited adaptation to local conditions, even suggesting they may seek to exert consumer-country control over the coffee supply chain. (Bose et al. 2016; Mithöfer et al. 2018). Although producer cooperatives represent a locally led governance structure, some farmers have expressed concerns at not being adequately engaged during cooperative decision-making processes (Hernandez-Aguilera et al. 2018). Thus, power asymmetry should be addressed at all stages of the coffee supply chain if these initiatives are to become truly sustainable.

In the Brazilian government “Rio Rural” programme the coffee-producing community is directly involved in the implementation of the initiative through collecting data and developing suggestions to address the sustainability challenges they encounter (Morais and César Pinheiro Da Silva 2021). Through such participatory approaches, it becomes possible for local actors to design and implement their own sustainability initiatives. The support of an active government department and appropriate policy in this case highlights the importance of collaboration and creating an enabling environment for coffee producers to pursue their visions of sustainability.

### Sustainability governance

By classifying coffee sector sustainability initiatives according to the scale of the agents designing the initiative, we identified a bias in research focusing on initiatives governed by distant actors. Certifications and other distant initiatives may suffer from a type of social-ecological scale mismatch, wherein the capacity and expertise of the institutions aspiring to manage global coffee sustainability do not match the local scale of decisions and behaviours that collectively impact sustainability of the sector (Cumming et al. 2006). These global-scale initiatives provide a degree of flexibility and adaptability, but may suffer from a lack of specific knowledge regarding the local context and challenges (Bose et al. 2016). Such “one size fits all” approaches to environmental governance have been shown to be relatively ineffective, and yet they remain prioritized over local, decentralized approaches to governance, such as the examples we provide here (Ostrom 2010).

Local-scale initiatives can overcome this discrepancy by drawing on local knowledge and contexts to develop local solutions, representing a case in which coffee producers drive *their own agenda* for change, rather than being forced to respond to an externally imposed agenda. Authors have highlighted that there is little research examining the contributions of local initiatives to sustainability transitions (Bennett et al. 2017; Lam et al. 2022) and that impact studies in the coffee sector need to move beyond certifications to investigate a broader suite of approaches (Millard 2017). Given that equity is now a key focus for sustainability transitions (Temper et al. 2018), it is important that future research investigates novel, locally led sustainability initiatives and how well certifications recognize and reward these initiatives.

It has been hypothesized that there are four major characteristics required for successful transformative governance for biodiversity, namely that initiatives need to be integrative, adaptive, pluralist and inclusive (Visseren-Hamakers et al. 2021). Local-scale initiatives in the coffee sector potentially exhibit three of these characteristics; they are inclusive, empowering smallholders whose interests may previously have been overlooked; adaptive in that they allow learning and responses which suit the local context, and pluralist through their recognition and application of diverse knowledge systems. A missing element might relate to their integration, by way of learning and knowledge sharing that can subsequently encourage the development of other local-scale solutions. In addition, monetizing or capitalizing on the local-scale initiatives via local and global markets could further improve sustainability.

### Partnerships for change

We documented several interactions among sustainability initiatives in the coffee supply chain. These interactions highlight how multiple actors, dispersed across various stages and scales of the supply chain are increasingly collaborating to address sustainability challenges. Producer cooperatives were often key to interactions, acting as the institution or initiative through which coffee producers obtain certification for their coffee, and facilitating access to required financial or other resources (Walenta 2015; Mili et al. 2019). Roasters implementing the relationship coffee model (RCM) also rely on certifications to inform purchasing decisions in some instances and may form partnerships with cooperatives in others (Simpson and Rapone 2000). A limitation with the locally led approaches is that smallholder farmers lack the resources to make these transitions themselves (Le et al. 2021), particularly as coffee yields can decline at first when they cease to use agrochemicals, before subsequently recovering as natural fertilizers and pest control take effect (Häger et al. 2021). This suggests that partnerships with NGOs and government agencies may be critical in assisting smallholders to overcome these barriers and make the transition towards more sustainable farming (Prihayati and Veriasa 2021). Future research could examine examples of government policies supporting grassroots initiatives to determine how best to structure such support.

Our analysis also revealed how coffee producers are applying a diversity of tools, with one case from Costa Rica including a family-owned farm which, due to concerns over pollutants and human health on the farm, has undergone a conversion back to traditional farming by applying agroecology principles (Häger et al. 2021). This producer used that transition back to traditional farming approaches to obtain organic certification, supported by their temporary participation in a cooperative, and subsequently established a direct-trade relationship (RCM) with a coffee buyer, and also used coffee tourism to generate further income to support their resilience. In this way they applied five different initiatives in tandem to improve their environmental, social, and economic sustainability.

### Further research gaps

Our review identified discrepancies between the volume of coffee that countries produced, and the amount of research attention that they had received. Mexico has been a geographic centre for research on sustainability initiatives, whilst their total volume of production is lower than other countries such as Brazil, Vietnam, Indonesia, and Ethiopia (SI 7). This mismatch may be due to factors related to research logistics such as ease of travel, local research capacity, and existing state partnerships that facilitate research. As the second largest coffee producer, Vietnam has received far less research attention to date, and West African countries even less still, although these countries produce smaller volumes of coffee. Both Vietnam and West Africa correspond with *robusta* coffee growing territories, and climate modelling suggests that this coffee variety will be more resilient to future changes (Bunn et al. 2015). The environmental sustainability of *arabica* producing systems is studied more than *robusta* systems, potentially as *arabica* has specialty markets while *robusta* is primarily traded as an undifferentiated commodity crop. However, it is important that future research investigate sustainability initiatives in these less well-documented countries and coffee species.

There was a noticeable gap in research focused on global multi-actor initiatives, which were represented by only two papers in our dataset. Both of these studies examined “4C”, or the “Common Code for the Coffee Community” (Manning and von Hagen 2010). 4C is variously described as both a multistakeholder initiative and a certification and is different to initiatives such as the Global Coffee Platform and Sustainable Coffee Challenge, which seek to galvanize stakeholders across the global supply chain. These programmes attempt to garner commitments to action from many actors around the world, leveraging significant financial resources, and as such it is imperative that their outcomes be examined (Millard 2017). The lack of research for this initiative type likely reflects their very recent appearance in the coffee sector; most of these have only been developed in the last five to ten years. The complexity of these initiatives, which may include thousands of different actors at multiple scales and stages of the supply chain, will limit the kinds of research which may be undertaken. Of particular importance will be assessing whether such commitments lead to tangible changes for the actors involved, and clarifying the theory of change by which these large-scale initiatives intend to act.

Our analysis of outcomes highlighted an overall paucity of knowledge regarding which sustainability initiatives most effectively deliver positive outcomes. We thus suggest that future research focus on assessing outcomes where possible, and that it do so via the lens of theories of change, to better understand how these initiatives are structured and intend to deliver outcomes (Rice et al., 2020). Examination of the diverse theories of change and the assumptions underpinning each initiative type is also essential information to support their implementation and improvement. Understanding the motivations of actors participating in these initiatives and drawing from disciplines such as conservation social science is another approach which could advance the field (Dayer et al. 2020). Where possible, undertaking comparative research of different initiatives in similar contexts can also help us to understand which are most effective at delivering sustainability.

## Conclusion

The diversity of sustainability initiatives in the coffee sector, and the broad suite of actors, scales, and approaches, demonstrates that coffee is on a journey towards sustainability. But the critical question remains as to how progress towards sustainability can be adequately measured and tracked. Whilst scientific research has focused on the outcomes of certifications and much attention has been given to their impact, there is a broader suite of interventions that require investigation. Additionally, environmental outcomes should be framed as a key consideration for sustainability initiatives, and we suggest that more research explicitly tests the environmental impacts of sustainability initiatives. Locally led initiatives, driven by coffee farmers themselves in response to social–ecological challenges, may represent an example of an inclusive sustainability transition and require further investigation, to broaden our understanding of the diverse pathways towards sustainability.

### Supplementary Information

Below is the link to the electronic supplementary material.

Supplementary file 1 (PDF 260 kb)

## References

[CR1] Altieri MA (2002). Agroecology: The science of natural resource management for poor farmers in marginal environments. Agriculture, Ecosystems & Environment.

[CR2] Altieri MA, Toledo VM (2011). The agroecological revolution in Latin America: Rescuing nature, ensuring food sovereignty and empowering peasants. Journal of Peasant Studies.

[CR3] Bacon CM, Mendez VE, Gliessman SR, Goodman D, Fox JA (2008). Confronting the Coffee Crisis: Fair Trade, Sustainable Livelihoods and Ecosystems in Mexico and Central America.

[CR4] Bager SL, Lambin EF (2020). Sustainability strategies by companies in the global coffee sector. Business Strategy and the Environment.

[CR5] Bennett NJ, Roth R, Klain SC, Chan K, Christie P, Clark DA, Cullman G, Curran D (2017). Conservation social science: Understanding and integrating human dimensions to improve conservation. Biological Conservation.

[CR6] Bose A, Vira B, Garcia C (2016). Does environmental certification in coffee promote “business as usual”? A case study from the Western Ghats, India. Ambio.

[CR70] Bro AS, Ortega DL, Clay DC, Richardson RB (2020). Understanding individuals’ incentives for climate change adaptation in Nicaragua's coffee sector. Climate and development.

[CR7] Bunn C, Läderach P, Ovalle Rivera O, Kirschke D (2015). A bitter cup: Climate change profile of global production of Arabica and Robusta coffee. Climatic Change.

[CR8] Candelo E, Casalegno C, Civera C, Büchi G (2019). A ticket to coffee: Stakeholder view and theoretical framework of coffee tourism benefits. Tourism Analysis.

[CR9] Castro LM, Calvas B, Hildebrandt P, Knoke T (2013). Avoiding the loss of shade coffee plantations: How to derive conservation payments for risk-averse land-users. Agroforestry Systems.

[CR10] Coffey, B., F.L.P. Damiens, E. Hysing, and N. Torabi. 2022. Assessing biodiversity policy designs in Australia, France and Sweden. Comparative lessons for transformative governance of biodiversity? 10.1080/1523908X.2022.2117145.

[CR11] Covidence. (2022) Covidence systematic review software, Veritas Health Innovation, Melbourne, Australia. Available at www.covidence.org.

[CR12] Cumming GS, Cumming DHM, Redman CL (2006). Scale mismatches in social-ecological systems: Causes, consequences, and solutions. Ecology and Society.

[CR13] Dayer AA, Silva-Rodríguez EA, Albert S, Chapman M, Zukowski B, Ibarra JT, Gifford G, Echeverri A (2020). Applying conservation social science to study the human dimensions of Neotropical bird conservation. The Condor.

[CR14] DeFries, R.S., J. Fanzo, P. Mondal, R. Remans and S.A. Wood. 2017. Is voluntary certification of tropical agricultural commodities achieving sustainability goals for small-scale producers? A review of the evidence. *Environmental Research Letters*. 10.1088/1748-9326/aa625e.

[CR75] Dietz T, Grabs J, Chong AE (2021). Mainstreamed voluntary sustainability standards and their effectiveness: Evidence from the Honduran coffee sector. Regulation & Governance.

[CR15] Edelmann H, Quiñones-Ruiz XF, Penker M (2022). How close do you like your coffee? Examining proximity and its effects in relationship coffee models. Journal of Rural Studies.

[CR16] FAO. 2014. *Safa Sustainability Assessment of Food and Agriculture Systems Guidelines*.

[CR17] Francis, J., and R.-D. Hyman. 2013. The impact of geographical indications on the economic, cultural, social, and environmental pillars of sustainability: The Case Study of Jamaican Blue Mountain Coffee. *The International Journal of Social Sustainability in Economic, Social and Cultural Context*, *8*.

[CR18] Garrett RD, Levy SA, Gollnow F, Hodel L, Rueda X (2021). Have food supply chain policies improved forest conservation and rural livelihoods? A systematic review. Environmental Research Letters.

[CR19] Grabs J, Ponte S (2019). The evolution of power in the global coffee value chain and production network. Journal of Economic Geography.

[CR20] Grames EM, Stillman AN, Tingley MW, Elphick CS (2019). An automated approach to identifying search terms for systematic reviews using keyword co-occurrence networks. Methods in Ecology and Evolution.

[CR21] Haddaway NR, Macura B, Whaley P, Pullin AS (2018). ROSES Reporting standards for Systematic Evidence Syntheses: Pro forma, flow-diagram and descriptive summary of the plan and conduct of environmental systematic reviews and systematic maps. Environmental Evidence.

[CR22] Haddaway NR, Bethel A, Dicks LV, Koricheva J, Macura B, Petrokofsky G, Pullin AS, Savilaakso S (2020). Eight problems with literature reviews and how to fix them. Nature Ecology and Evolution.

[CR23] Häger A, Little M, Amel E, Calderón G (2021). Transformation toward sustainability on a Costa Rican coffee farm: Environmental, socioeconomic, and psychological perspectives. Case Studies in the Environment.

[CR24] Hernandez-Aguilera JN, Gómez MI, Rodewald AD, Rueda X, Anunu C, Bennett R, van Es HM (2018). Quality as a driver of sustainable agricultural value chains: The case of the relationship coffee model. Business Strategy and the Environment.

[CR25] Hoang, V., and A. Nguyen. 2019. PGI Buon Ma tuot coffee in Vietnam. *Sustainability of European Food Quality Schemes: Multi-Performance, Structure, and Governance of PDO, PGI, and Organic Agri-Food Systems*, 265–285. 10.1007/978-3-030-27508-2_14.

[CR26] ICO. 2014. World coffee trade (1963–2013): A review of the markets, challenges and opportunities facing the sector. *Reviev of the 112th International Coffee Council*, *February*, 29. www.ico.org.

[CR71] International Cooperative Alliance. 2023. https://ica.coop/en/cooperatives/cooperativeidentity.

[CR27] Lam DPM, Jiménez-Aceituno A, Guerrero Lara L, Sellberg MM, Norström AV, Moore ML, Peterson GD, Olsson P (2022). Amplifying actions for food system transformation: Insights from the Stockholm region. Sustainability Science.

[CR28] Lara-Estrada L, Rasche L, Schneider UA (2021). Land in Central America will become less suitable for coffee cultivation under climate change. Regional Environmental Change.

[CR29] Le QV, Jovanovic G, Le DT, Cowal S (2020). Understanding the perceptions of sustainable coffee production: A case study of the k’ho ethnic minority in a small village in Lâm Dông Province of Vietnam. Sustainability.

[CR30] Le QV, Cowal S, Jovanovic G, Le DT (2021). A study of regenerative farming practices and sustainable coffee of ethnic minorities farmers in the central highlands of Vietnam. Frontiers in Sustainable Food Systems.

[CR31] Manning S, von Hagen O (2010). Linking local experiments to global standards: How project networks promote global institution-building. Scandinavian Journal of Management.

[CR72] Marie-Vivien D, Garcia CA, Kushalappa CG, Vaast P (2014). Trademarks, geographical indications and environmental labelling to promote biodiversity: The case of agroforestry coffee in India. Development Policy Review.

[CR32] Martínez NM (2016). Towards a network place branding through multiple stakeholders and based on cultural identities: The case of “The Coffee Cultural Landscape” in Colombia. Journal of Place Management and Development.

[CR33] Meemken, E.M. 2020. Do smallholder farmers benefit from sustainability standards? A systematic review and meta-analysis. *Global Food Security*. 10.1016/j.gfs.2020.100373

[CR73] Melo Torres LI, Melo Torres MM, Fonseca Pinto DE (2017). The Associativity: a local development strategy for Ocamonte (APCO) coffee growers in Santander. Colombia. Acta Agronómica.

[CR34] Microsoft Corporation. 2018. *Microsoft Excel*. Retrieved from https://office.microsoft.com/excel.

[CR35] Milder JC, Arbuthnot M, Blackman A, Brooks SE, Giovannucci D, Gross L, Kennedy ET, Komives K (2014). An agenda for assessing and improving conservation impacts of sustainability standards in tropical agriculture. Conservation Biology.

[CR36] Mili, S., C. Ferro Soto, and A. Bouayad. 2019. Measuring the overall and integrated performance of socially responsible companies: The case of fair trade. In *International Journal of Engineering and Advanced Technology (IJEAT)* (Issue 3).

[CR37] Millard E (2017). Still brewing: Fostering sustainable coffee production. World Development Perspectives.

[CR38] Mithöfer D, Méndez VE, Bose A, Vaast P (2018). Harnessing Local Strength for Sustainable Coffee Value Chains in India and Nicaragua: Reevaluating Certification to Global Sustainability Standards..

[CR39] Morais MO, Da Silva  A César Pinheiro  (2021). Political architectures in the municipality of Varre-Sai (Brazil): For sustainabilities in ‘specialty coffees’’ production management’. Royal Society Open Science.

[CR40] Newton P, Agrawal A, Wollenberg L (2013). Enhancing the sustainability of commodity supply chains in tropical forest and agricultural landscapes. Global Environmental Change.

[CR41] Ostrom E (2010). Beyond markets and states: Polycentric governance of complex economic systems. American Economic Review.

[CR42] Panhuysen, S., and J. Pierrot. 2020. *Coffee Barometer*.

[CR43] Prihayati Y, Veriasa TO (2021). Developing green tourism to create the sustainable landscape: Evidence from Community-based Coffee Tourism (CbCT) in Puncak, Bogor, Indonesia. IOP Conference Series: Earth and Environmental Science..

[CR44] Pronti A, Coccia M (2021). Multicriteria analysis of the sustainability performance between agroecological and conventional coffee farms in the East Region of Minas Gerais (Brazil). Renewable Agriculture and Food Systems.

[CR45] QSR International Pty Ltd. 2018. NVivo (Version 12), https://www.qsrinternational.com/nvivo-qualitative-data-analysis-software/home.

[CR46] R Core Team. 2021. R: A language and environment for statistical computing. R Foundation for Statistical Computing, Vienna, Austria. https://www.R-project.org/.

[CR67] Rice WS, Sowman MR, Bavinck M (2020). Using Theory of Change to improve post-2020 conservation: A proposed framework and recommendations for use. Conservation Science and Practice.

[CR47] Richardson, K., W. Steffen, W. Lucht, J. Bendtsen, S.E. Cornell, J.F. Donges, and J. Rockström. 2023. Earth beyond six of nine planetary boundaries. *Science Advances*, *9*, eadh2458. 10.1126/sciadv.adh2458 PMC10499318 37703365

[CR48] Rockström J, Edenhofer O, Gaertner J, DeClerck F (2020). Planet-proofing the global food system. Nature Food.

[CR49] Rockström, J., W. Steffen, K. Noone, Å. Persson, F.S. Chapin, E. Lambin, T.M. Lenton, M. Scheffer, et al. 2009. Planetary boundaries: Exploring the safe operating space for humanity. *Ecology and Society*. 10.5751/ES-03180-140232.

[CR50] Rueda X, Garrett RD, Lambin EF (2017). Corporate investments in supply chain sustainability: Selecting instruments in the agri-food industry. Journal of Cleaner Production.

[CR51] Sachs JD, Cordes K, Rising J, Toledano P, Maennling N (2020). Ensuring economic viability and sustainability of coffee production. SSRN Electronic Journal.

[CR52] Samper LF, Quiñones-Ruiz XF (2017). Towards a balanced sustainability vision for the coffee industry. Resources.

[CR53] Schneider A, Sidney M (2009). What is next for policy design and social construction theory?. Policy Studies Journal.

[CR54] Simpson, C.R., and A. Rapone. 2000. Community development from the ground up: Social-justice coffee. In *Source: Human Ecology Review* (Vol. 7, Issue 1).

[CR55] Smith BG (2007). Developing sustainable food supply chains. Philosophical Transactions of the Royal Society B: Biological Sciences.

[CR56] Stiglitz, J., A. Sen, and J. Fitoussi. 2009. *Report by the Commission on the Measurement of Economic Performance and Social Progress*. https://www.cbs.nl/-/media/imported/documents/2011/36/stiglitzsenfitoussireport_2009.pdf

[CR77] Sustainable Coffee Challenge. 2020. https://www.sustaincoffee.org/

[CR66] Taplin DH, Clark H (2012). Theory of change basics: A primer on theory of change. New York NY: ActKnowledge.

[CR76] Taringana T, Mtisi JP (2019). The Sustainability of Rural Livelihoods and Ecology among Smallholder Coffee Farmers in the Eastern Districts of Zimbabwe, 1980–2018. Global Environment.

[CR57] Tayleur C, Balmford A, Buchanan GM, Butchart SHM, Corlet Walker C, Ducharme H, Green RE, Milder JC (2018). Where are commodity crops certified, and what does it mean for conservation and poverty alleviation?. Biological Conservation.

[CR58] Temper L, Walter M, Rodriguez I, Kothari A, Turhan E (2018). A perspective on radical transformations to sustainability: Resistances, movements and alternatives. Sustainability Science.

[CR59] Traldi R (2021). Progress and pitfalls: A systematic review of the evidence for agricultural sustainability standards. Ecological Indicators.

[CR60] Urgessa Waktola T, Fekadu K (2021). Adoption of coffee shade agroforestry technology and shade tree management in Gobu Seyo District, East Wollega, Oromia. Advances in Agriculture.

[CR61] Visseren-Hamakers IJ, Razzaque J, McElwee P, Turnhout E, Kelemen E, Rusch GM, Fernández-Llamazares Á, Chan I (2021). Transformative governance of biodiversity: Insights for sustainable development. Current Opinion in Environmental Sustainability.

[CR62] Walenta J (2015). Becoming carbon neutral: Evaluating the carbon neutral certification as a tool for reducing climate change impacts and securing financial livelihoods. Sustainability (united States).

[CR63] Weber H, Wiek A (2021). Cooperating with “Open Cards”—The role of small intermediary businesses in realizing sustainable international coffee supply. Frontiers in Sustainable Food Systems.

[CR64] Westgate MJ (2019). revtools: An R package to support article screening for evidence synthesis. Research Synthesis Methods.

[CR65] Yudhari IDAS, Darwanto DH, Waluyati LR, Mulyo JH (2020). Multidimensional Scaling: Sustainability of Arabika Coffee Agro-Tourism in Kabupaten Bangli Bali. Journal of Environmental Management and Tourism.

