# Supplementary material for: Sustainable coffee: A review of the diverse initiatives and governance dimensions of global coffee supply chains
Source: Ambio. 2024 Apr 29;53(7):984–1001. doi: 10.1007/s13280-024-02003-w (PMC11101400; doi:10.1007/s13280-024-02003-w)

Ambio

Electronic Supplementary Material

This supplementary information has not been peer reviewed.

Title: **Sustainable coffee: A review of the diverse initiatives and governance dimensions of global coffee supply chains.**

Authors: Wright, D.R., Bekessy, S.A., Lentini, P.E., Garrard, G.E., Gordon, A., Rodewald, A.D., Bennett, R.E., Selinske, M.J.

SI 1. Extensive initial list of synonyms generated from pilot search.

Coffee supply chain	Sustainability instrument	Relationship coffee model	Governance	Commodity round-table	Ban	Geographic indication
Coffee value chain	Sustainability intervention	Direct investment at origin	Sustainability governance	Multi-stakeholder initiative	Moratorium	Appellation of origin
Global value chain	Sustainability strategy	Direct trade model	Environmental governance	Multi-stakeholder instrument		
Global supply chain	Sustainability practice		Socio-economic practices	Corporate social responsibility		
Sustainable supply chain	Sustainability standard		Environmental practices	Internal code of conduct		
Supply chain policy	Voluntary sustainability standard		Environmental policy			
Supply chain resilience	certification					
	End-market standard					
Supply chain vulnerability	Market-based mechanism					

Naïve search string:

coffe* AND (biodiver* OR sustainab* OR environ*) AND ("supply chain*" OR certificat* OR governance OR “value chain*” OR polic* OR practice* OR instrument* OR "corporate social responsibility")

Litsearchr process

Litsearchr generates candidate lists of keywords by either extracting author tagged keywords (n=1654) or identifying common terms from the title and abstract of each manuscript (“raked keywords” = 12915). We applied both methods and combined the results to identify 13 470 unique keywords. A keyword co-occurrence network is used to estimate the strength of each term, given how many terms with which it co-occurs in a manuscript. A cut-off point of 3 was applied to generate a final list of the strongest keywords in the network, resulting in 64 keywords. The package then processes these keywords to produce a draft search string. The keyword list and draft search string was manually inspected to identify additional terms for inclusion in the search string.

Litsearchr keyword list:

1] "age" "area" **"coffee"** "diversity"

[5] "**environment" "environmental" "farm" "farmers"**

[9**] "forest" "impact**" "land" "management"

[13] "market" "part" "plant" "product"

[17] "production" "search" "**social" "sustainable"**

[21] "system" "systems" "ability" "**actor**"

[25] "agricultural" "analysis" "approach" "based"

[29] "cation" "change" "count" "**cultural**"

[33] "develop" "differ" **"economic"** "effect"

[37] "farmer" "import" "increase" "inter"

[41] "logic" "logical" "manag" "manage"

[45] "mental" "nation" "potential" **"practice"**

[49] "**practices"** "present" "produce" "prove"

[53] "provide" "ratio" "research" "result"

[57] "results" "serve" "significant" "small"

[61] "stand" "stems" "study" "**sustain"**

SI 2. Number of manuscripts extracted

Database	Output
Web of Science	765 results
Scopus	780 results
ProQuest	589 results
**Initial Dataset**	**2134 results**
Duplicate Records - extracted	908 results
**Dataset for screening**	**1226 results**

SI 3. Exclusion criteria.

- Exclude studies which do not include coffee.
- Exclude pure ecology or agriculture studies. This subset included papers: examining ecosystem services, local ecological knowledge for agriculture, land use change, deforestation and land use planning studies, remote sensing or GIS studies, climate change and coffee distribution modelling studies.
- Exclude food science, coffee quality, plant breeding and genetics studies (dietary analysis).
- Exclude marketing, branding and consumer psychology studies.
- Exclude studies on human health dimensions of coffee (or other human health studies).
- Exclude studies examining biomass valorization in the coffee supply chain.
- Political science studies (ie. agrarian movements, philosophy).
- Exclude information systems, computer science or design studies (manufacturing / engineering studies).

SI 4. Additional exclusion criteria

- Exclude papers which do not include one or more sustainability initiatives (as per our definition)
- Exclude studies which consider multiple commodities.
- Exclude general governance studies or landscape level studies not focused on coffee specifically
- The review focused on initiatives working on the production side of the supply chain.
- Excluded blockchain technology and circular economy studies

SI 5.

Please contact the lead author for the full manuscript dataset.

SI 6. Terms in use for coffee sustainability initiatives.

These terms were documented during the eligibility screening stage of the review and subsequently refined to produce the typology.

**Community & Cultural Initiatives**	**Producer Cooperative**	**Agroecology programs**	**Climate–smart agriculture**	**Geographical Indications**	**Payments for Ecosystem Services**	**Government Policy**	**Relationship Coffee Model**	**Corporate Social Responsibility Initiative**	**Certifications**	**Global Multi-actor initiatives**
Coffee cultural landscape	Local / Traditional Institution	Agroforestry price support	Carbon neutral certification	Appellation of origin	Conservation payments		Direct-trade model	Private sustainability standards	Voluntary sustainability standards	Collaborative Industry Platforms
Community-based coffee tourism	Social Entrepreneurs	Agroforestry incentives	Product carbon footprint	Denomination of origin	Conservation fees		Contract Farming	In-house certifications	Market-based instrument	4C – Common Code for Coffee Initiative
Community-based Forest management		Agroecology policy	Environmental footprint	Environmental labelling			Mediated Partnership Model	Internal Company Policy	Alternative Trade Organisation (FTO)	*Global Coffee Platform*
Property Rights			Water footprint				Social Brokerage	*Nespresso AAA**	Price premiums	*Sustainable Coffee Challenge*
Tenure agreements							Specialty Coffee Markets	*Starbucks CAFÉ**	Eco-labelling	
Socio-cultural benefits										

SI 7. Figure showing the volume of coffee production in 2020 versus the number of studies in our dataset.

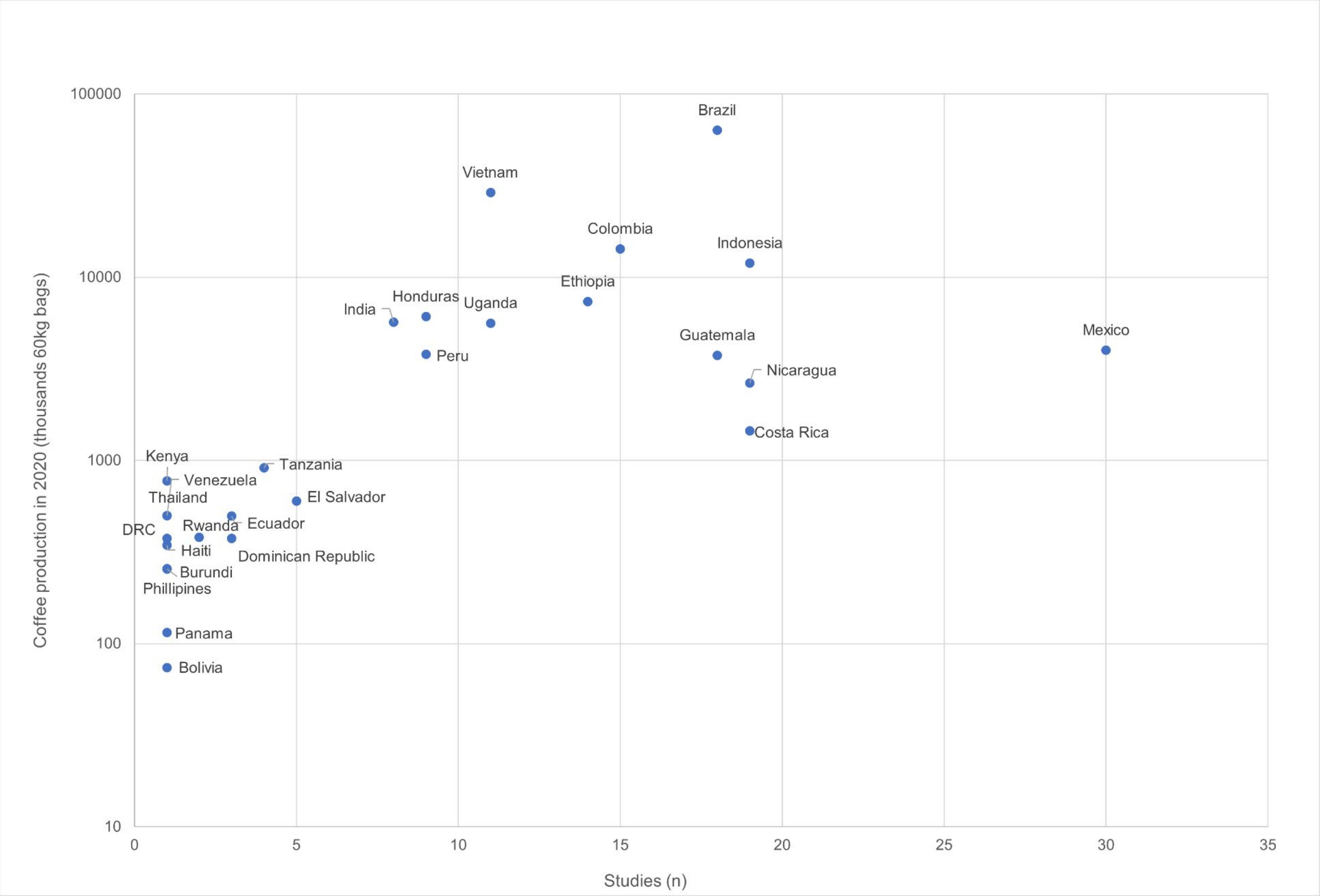

Supplement: Supplementary file 1 — (PDF 260 kb) [file 13280_2024_2003_MOESM1_ESM.pdf]
